# Laparoscopic proximal gastrectomy with right-sided overlap and single-flap valvuloplasty (ROSF): a case-series study

**DOI:** 10.1186/s12893-023-01975-y

**Published:** 2023-04-14

**Authors:** Wei Peng, Shangcheng Yan, Yikai Huang, Ming Cheng, Tianhua Liu, Rui Ren, Qiang Chen, Jingyu Zhang, Wei Gong, Chungen Xing, Yongyou Wu

**Affiliations:** Department of Gastrointestinal Surgery, Second Affiliated Hospital of Soochow University, Suzhou, China

**Keywords:** Esophagogastrostomy, Laparoscopy, Proximal gastrectomy, Esophagogastric junction, Gastric cancer

## Abstract

**Background:**

There is no standard reconstruction method following proximal gastrectomy, of which gastroesophageal reflux and anastomotic complications are of great concern. Though several techniques have been devised to overcome these postoperative complications, such as double tract reconstruction, double-flap technique and side overlap fundoplication by Yamashita, none of them is considered a perfect solution. Herein, we designed a novel method of esophagogastrostomy after laparoscopic proximal gastrectomy (LPG), named right-sided overlap and single-flap valvuloplasty (ROSF).

**Methods:**

Between March 2021 and December 2021, 20 consecutive patients underwent LPG-ROSF at Department of Gastrointestinal Surgery, Second Affiliated Hospital of Soochow University. Surgical outcomes and postoperative complications were recorded. All patients were followed-up until December 2022. Endoscopy and assessment of gastrointestinal symptoms were performed 1 year after surgery. Nutrition-related parameters including total body weight, hemoglobin, lymphocyte count, serum total protein, serum albumin and serum prealbumin were evaluated 1 year after surgery and compared with those before surgery.

**Results:**

The mean surgery time and anastomosis time was 285.3 ± 71.3 and 61.3 ± 11.2 min respectively. None of the patients had gastrointestinal early postoperative complications. Symptomatic reflux was observed in one patient (5%) while reflux esophagitis (Los Angeles Grade A) was observed in another patient (5%). Four patients (20%) had mild dysphagia (Visick score = II) but none of them had anastomotic stenosis. There were no significant changes in nutritional status postoperatively.

**Conclusions:**

ROSF can be safely performed after LPG and has satisfactory outcomes in preventing reflux and stenosis, and maintaining nutritional status. This technique requires further validation.

**Supplementary Information:**

The online version contains supplementary material available at 10.1186/s12893-023-01975-y.

## Background

Though the incidence of gastric cancer is declining worldwide, the proportion of lesions located in the upper-third of the stomach and esophagogastric junction (EGJ) is increasing [1]. For such cases, total gastrectomy (TG) is still performed regardless of TNM stages in some institutions, leading to impaired quality of life (QOL), mainly presenting with severe weight loss and decreased hemoglobin [2]. Owing to dramatic progress made in endoscopic diagnosis, the ratio of early gastric cancer (EGC) is climbing in China [3]. For some EGC in the upper-third of the stomach and EGJ, TG is unnecessary for mere oncological purposes. Even for advanced cancer at the EGJ with a diameter less than 4 cm, metastasis in the lymph nodes (LNs) along the distal part of the stomach is extremely rare and proximal gastrectomy (PG) can be performed safely without impairing oncological outcomes [4].

However, the risk of reflux is a great concern after PG. Several techniques have been devised to overcome postoperative esophageal reflux, among which, esophagogastrostomy with double-flap technique (DFT) is proved to be satisfactory with respect to its anti-reflux effect [5]. However, this procedure is extremely technically demanding and time-consuming, with certain incidences of complications, such as stricture and failure of anastomosis [5, 6]. Side overlap fundoplication by Yamashita (SOFY) is a relatively simple method, but the anti-reflux effect varies among individuals and may be worse than DFT [7].

To overcome the shortcomings of the existent anti-reflux esophagogastrostomies, we designed a novel esophagogastrostomy method following laparoscopic proximal gastrectomy (LPG), namely right-sided overlap and single-flap valvuloplasty (ROSF). Herein, we present the clinical outcomes of the initial 20 cases.

## Methods

### Patients

This is a retrospective case-series study of 20 consecutive patients who underwent LPG-ROSF at Second Affiliated Hospital of Soochow University, Suzhou, China, between March 2021 and December 2021. The study was approved by The Ethics Committee of Second Affiliated Hospital of Soochow University. All patients and their families were informed of the novel technique preoperatively and signed their consent.

LPG-ROSF was indicated for patients diagnosed with cT1-2N0M0 adenocarcinoma located at EGJ or upper-third stomach. All diagnoses were confirmed preoperatively by endoscope, biopsy and contrast-enhanced computed tomography (CECT). Preservation of at least half of the stomach and R0 resection were prerequisite for our procedure. Patients did not have severe comorbidities with American Society of Anesthesiologists Physical Status (ASA-PS) ≤ 2. Patients with EGC were indicated only when endoscopic submucosal dissection (ESD) was considered unindicated by multidisciplinary treatment team or ESD failed to achieve R0 resection.

### Surgical procedure

#### Laparoscopic proximal gastrectomy

**Fig. 1:**
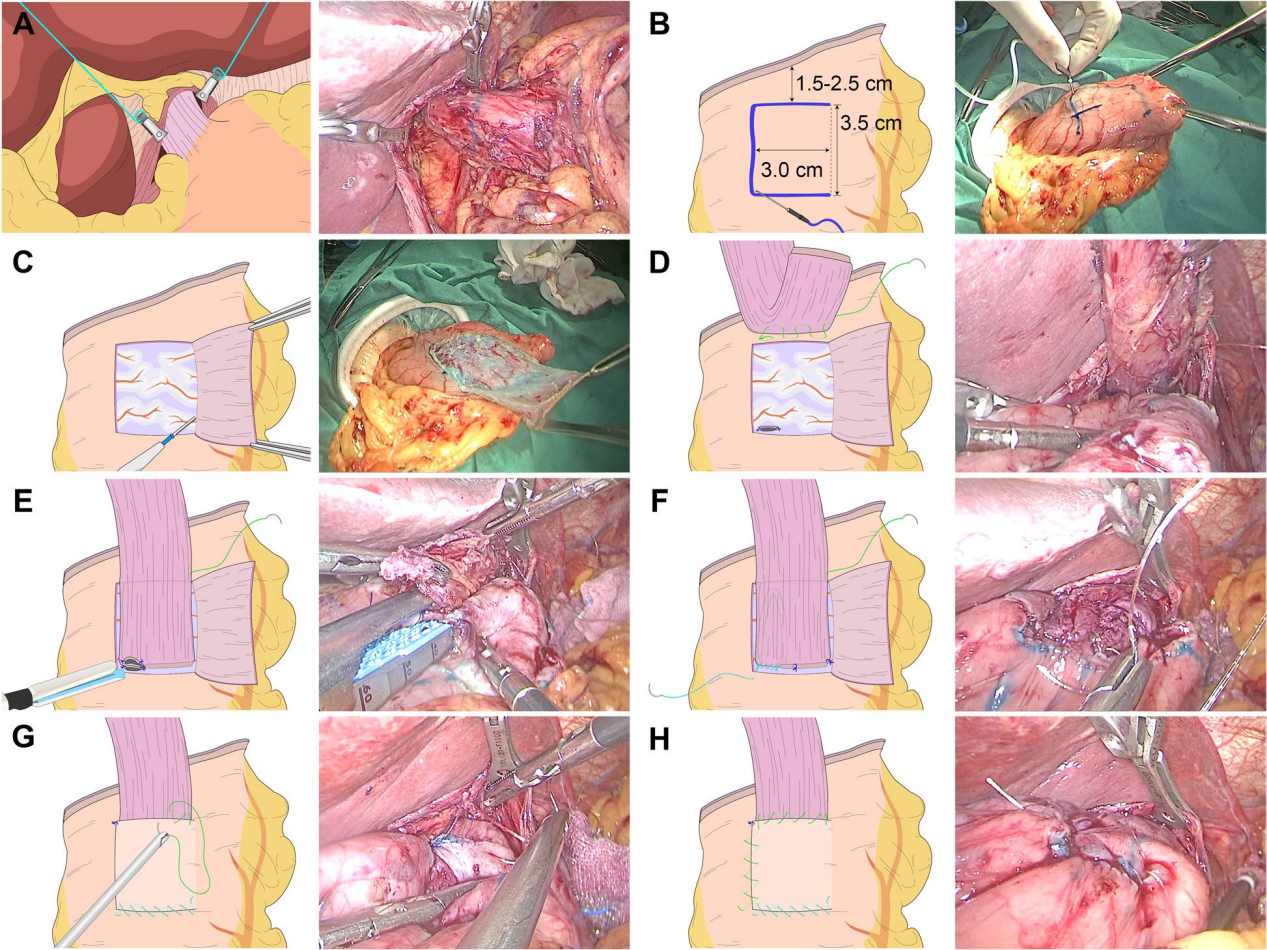
Surgical procedure of right-sided overlap and single-flap valvuloplasty. **A** Two internal organ retractors were clipped to right and left diaphragmatic crura. **B** A 3.0 × 3.5 cm “匚”-shaped region was marked with methylene blue, 1.5–2.5 cm distal to transection line of the stomach. Diluted methylene blue was injected submucosally. **C** A left-sided flap was made by cutting between submucosa and muscular propria with an electric scalpel. **D** Posterior esophageal wall was fixed with anterior gastric wall using a barbed suture. **E** A linear stapler was inserted into the openings of the stomach and esophagus to perform side overlap anastomosis. **F** A second barbed suture was used to close the common opening. The lower edge of the esophageal stump was fixed by two interrupted sutures. **G** The distal brim of the flap was sutured using the second barbed suture. An interrupted suture anchored the flap on upper-right corner. The proximal and right brim of the flap were sutured using the first barbed suture. **H** Final shape of the single flap

A 10–12 mmHg pneumoperitoneum was created by the injection of carbon dioxide, and five trocars of 5 or 12 mm were inserted. After laparoscopic exploration excluding serosal invasion and distant metastasis, LPG and D2 lymphadenectomy was performed in accordance with Japanese Gastric Cancer Treatment Guidelines [8]. No. 1, 2, 3a, 4sa, 4sb, 7, 8a, 9, 11p and 11d LNs were dissected in all cases. No. 19, 20 and 110 LNs were dissected if the tumor involved the esophagus while No. 10 LN was dissected if the greater curvature was invaded. Surgery field of EGJ was exposed by two internal organ retractors clipped to right and left diaphragmatic crura (Fig. 1A). Two sutures were knotted to the clips intracorporeally, and were tensioned and fixed by hemostats extracorporeally. Phrenoesophageal ligament was resected to free the distal esophagus because a length of 5.0–6.0 cm is needed to complete anastomosis. Tumor location was marked preoperatively and confirmed by intraoperative endoscopy. After LN dissection, the esophagus was transected at least 2 cm proximal to the upper margin of the tumor by linear staplers. Intraoperative frozen biopsy was performed to guarantee a proximal negative margin. Transection of the stomach was performed extracorporeally through an epigastric midline incision. For early cases (cT1), the stomach was transected at least 2 cm distant to the lower edge of the lesion. For advanced cases (cT2), a distal margin of at least 5 cm was ensured. Frozen biopsy was performed if a safe distal margin was uncertain.

#### Esophagogastrostomy with ROSF

First, a single flap was prepared extracorporeally (Fig. 1B, C). A “匚”-shaped region with a width of 3.0 cm and height of 3.5 cm was marked on the anterior gastric wall with methylene blue, 1.5–2.5 cm distal to transection line of the remnant stomach. After submucosal injection of diluted methylene blue, a unilateral left-opening flap was made by carefully cutting between submucosa and muscular propria with an electric scalpel. A small incision was made at the right lower edge of the mucosal window for the following overlap anastomosis. Two sutures were placed on both ends of the incision to guide the stapler. The remnant stomach was put back into the abdominal cavity.

Then, the esophagus and stomach were anastomosed intracorporeally. The posterior wall of esophagus was fixed with the remnant stomach above the upper edge of the mucosal window by continuous suturing using a barbed suture (Fig. 1D). Length of the esophagus was measured by a ureteral catheter intracorporeally. The barbed suture was fastened and put aside for covering of the flap later. Next, an incision was made at the right lower edge of the esophageal stump. Forks of a 6-row linear stapler were inserted into the openings of the stomach and esophagus, stapling the mucosal window to the right side of the esophageal wall (Fig. 1E). The right sides of the esophagus and gastric mucosal window were stapled for 3.0 cm. Another barbed suture was used to close the common opening of the esophagus and the stomach. The lower edge of the esophageal stump was fixed to the mucosal window by two interrupted sutures (Fig. 1F).

Finally, the anastomotic site was covered with the single seromuscular flap (Fig. 1G, H). The distal brims of the mucosal window and the flap were sutured using the second barbed suture. An interrupted suture anchored the flap to the upper-right corner of the mucosal window. Using the first barbed suture, the proximal brim of the flap was sutured with the esophagus, and the right brim with the right brim of the mucosal window sequentially. After completion of anastomosis, a drainage tube was inserted trans-hiatally into the lower mediastinum, while nasogastric tube was not inserted routinely.

### Postoperative management

Patients were encouraged to restore ambulation and liquid diet on postoperative day (POD) 1. On POD 3–4, semi-liquid diet was restored. The draining tube was removed on POD 6–8. Iohexol contrast radiography was performed 1 month after surgery to evaluate anastomotic leakage and stenosis.

### Clinical analysis

Background characteristics including patients’ age, sex, ASA-PS, body mass index (BMI), history of preoperative ESD, postoperative pathology (depth of invasion, LN involvement, distant metastasis and pathological TNM stage) were obtained from electronic medical records. TNM stage was determined using American Joint Committee on Cancer Staging Manual (8th edition) [9]. Surgical outcomes were recorded, including surgery time, anastomosis time, estimated blood loss, number of retrieved LNs, time to first flatus and defecation, postoperative length of stay (LOS), 30-day rehospitalization, adjuvant therapy, recurrence and death. The anastomosis time was defined as time of extracorporeal flap preparation plus time of intracorporeal anastomosis.

Early and late postoperative complications were also recorded and evaluated using the Clavien-Dindo (CD) classification [10]. Early postoperative complications (occurred ≤ 30 days after surgery) include anastomotic leakage, intrabdominal bleeding, intestinal obstruction, pulmonary and cardiovascular complications. Late postoperative complications (occurred > 30 days after surgery) include anastomotic stenosis, reflux esophagitis, gastric residue and proton pump inhibitor (PPI) administration. Reflux esophagitis was assessed by endoscopy according to the Los Angeles (LA) Classification [11], and was recorded when LA grade ≥ A. Anastomotic stenosis was defined as having dysphagia associated with the inability to pass an 8-mm endoscope through the anastomosis as was described by Nishikawa et al. [12].

Patients were followed up every 3 months during the first postoperative year and every 6 months in the second postoperative year. All patients were followed-up until December 2022. Patients’ symptoms including heartburn, acid regurgitation, dysphagia, bloating, diarrhea and epigastric pain were inquired and evaluated using modified Visick score [13]. Total body weight, blood routine, serum biochemistry and tumor markers were measured at each outpatient visit and compared to the preoperative levels. Endoscopy was performed every year after surgery. CECT was performed every 6 months for Stage II patients and every 1 year for Stage I patients respectively.

### Statistical analysis

Values are presented as mean ± standard deviation for normally distributed continuous variables, as median (range) for discrete variables, and as number (%) for categorical variables. Nutrition-related parameters before surgery and at 1-year follow-up were compared using paired samples *t* test. A *p*-value < 0.05 was considered significant. Statistical analyses were performed using IBM Statistical Package for the Social Sciences version 25.0. Graphs were prepared in Adobe Illustrator version 25.2.1 and Adobe InDesign CC version 13.0.

## Results

### Patient characteristics

The clinicopathological characteristics of the patients are listed in Table 1. 18 patients were male (90%). The mean age was 67.2 ± 7.7 years. 15 patients (75%) had ASA-PS of 2. The mean preoperative BMI was 23.5 ± 3.1 kg/m^2^. Two patients (10%) received ESD preoperatively, which failed to achieve R0 resection. Postoperative pathology showed a mucosal (T1a) lesion in two, a submucosal (T1b) lesion in six, a T2 lesion in eight, and a subserosal (T3) lesion in four patients. LN involvement (N1) was observed in four patients (20%) and all involved LNs were on lesser curvature side. Distant metastasis was not observed in any patient. All patients had pathological stage (pStage) between IA and IIB.

**Table 1:** Clinicopathological characteristics of the patients

Variable	Value
Sex, n (%)	
Male	18 (90%)
Female	2 (10%)
Age (years), mean ± sd	67.2 ± 7.7
ASA-PS, n (%)	
1	5 (25%)
2	15 (75%)
BMI (kg/m^2^), mean ± sd	23.5 ± 3.1
Preoperative ESD, n (%)	2 (10%)
Depth, n (%)	
T1a	2 (10%)
T1b	6 (30%)
T2	8 (40%)
T3	4 (20%)
Lymph node metastasis, n (%)	
N0	16 (80%)
N1	4 (20%)
Distant metastasis, n (%)	
M0	20 (100%)
M1	0
pStage, n (%)	
IA	8 (40%)
IB	6 (30%)
IIA	4 (20%)
IIB	2 (10%)

*ASA-PS* American society of anesthesiologists physical status, *BMI* Body mass index, *ESD* Endoscopic submucosal dissection, *pStage* Pathological TNM stage

### Surgical results and postoperative complications

Surgical characteristics and postoperative complications are summarized in Table 2. The mean surgery time was 285.3 ± 71.3 min. The mean anastomosis time was 61.3 ± 11.2 min. The mean estimated blood loss was 59.0 ± 20.7 ml. The mean retrieved LNs number was 23.3 ± 8.9. The median time to first flatus and defecation was 2 (1–3) and 4 (2–9) days respectively. The median postoperative LOS after surgery was 11 (6–24) days. No patients were rehospitalized in 30 days after surgery. Six patients (20%) with pStage ≥ II received adjuvant chemotherapy postoperatively. No recurrence nor death was observed within the follow-up period.

**Table 2:** Surgical outcomes and postoperative complications

Variable	Value
Surgery time (min), mean ± sd	285.3 ± 71.3
Anastomosis time (min), mean ± sd	61.3 ± 11.2
Estimated blood loss (ml), mean ± sd	59.0 ± 20.7
Retrieved lymph nodes, mean ± sd	23.3 ± 8.9
Time to first flatus (days), median (range)	2 (1–3)
Time to first defecation (days), median (range)	4 (2–9)
Postoperative length of stay (days), median (range)	11 (6–24)
30-day rehospitalization, n (%)	0
Adjuvant therapy, n (%)	6 (30%)
Recurrence, n (%)	0
Death, n (%)	0
Early complications, n (%)	
Pulmonary	
CD Grade IIIa	1 (5%)
CD Grade II	1 (5%)
Cardiovascular	
CD Grade II	1 (5%)
Anastomotic leakage	0
Intrabdominal bleeding	0
Intestinal obstruction	0
Late complications, n (%)	
Anastomotic stenosis	0
Reflux esophagitis	
LA Grade A	1 (5%)
Gastric residue	0
PPI administration	2 (10%)

*CD* Clavien-Dindo, *LA* Los Angeles, *PPI* Proton pump inhibitor

Regarding early postoperative complications, anastomotic leakage, intrabdominal bleeding or intestinal obstruction were not observed. Pleural effusion was observed in two patients (10%), among which one received thoracentesis (CD Grade IIIa) while the other one was treated conservatively (CD Grade II). One patient (5%) had hypertensive emergency and recovered after medical treatment (CD Grade II).

Regarding late postoperative complications, no anastomotic stenosis was observed according to Nishikawa’s standard [12]. Endoscopy showed reflux esophagitis of LA Grade A in one patient (5%). Gastric residue was not found by endoscopy in any case. PPI was prescribed to two patients (10%) who had endoscopic or symptomatic reflux.

**Fig. 2:**
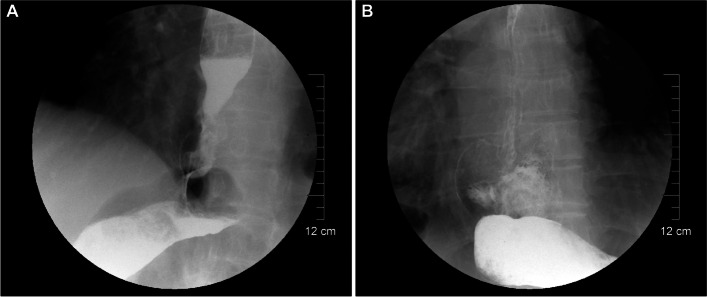
Iohexol contrast radiography performed 30 days after surgery. **A** Contrast passed through the anastomosis smoothly without leakage or stenosis. **B** Outline of the remnant stomach with a pseudofornix

Contrast radiography performed 30 days after surgery revealed no stenosis or leakage (Fig. 2A) and showed the outline of the remnant stomach with a pseudofornix (Fig. 2B).

**Fig. 3:**
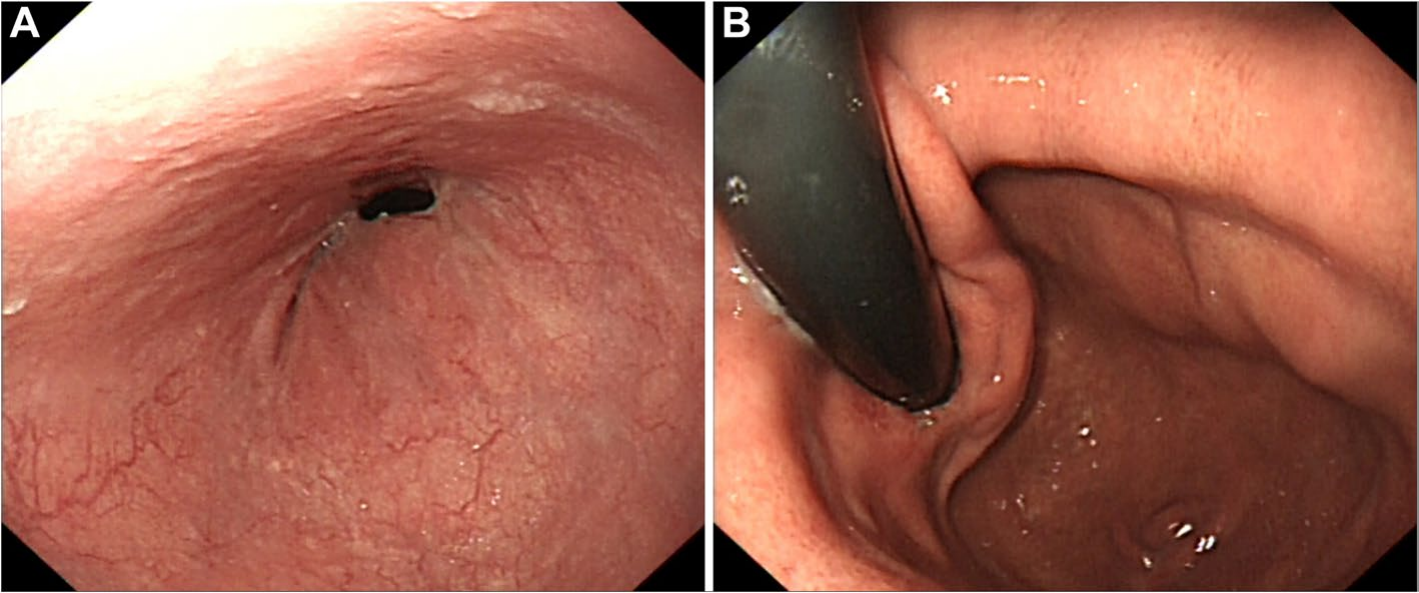
Endoscopic findings 1 year after surgery. **A** An oval-shaped anastomosis was observed while no erosion was observed in esophageal mucosa. **B** The angle of His and a pseudofornix had reformed

Figure 3 shows the endoscopic findings of a patient 1 year after LPG-ROSF. An oval-shaped anastomosis was observed while no erosion was observed in esophageal mucosa (Fig. 3A). Observation in the stomach showed the reformed angle of His and pseudofornix (Fig. 3B).

### Follow-up and postoperative nutritional status

The gastrointestinal symptoms and Visick scores 1 year after surgery are shown in Table 3. No patient reported heartburn and one patient (5%) reported mild acid regurgitation (Visick score = II). His symptom relieved after taking PPI. Four patients (20%) complained mild dysphagia (Visick score = II) but the symptoms could be avoided by swallowing slower or eating smaller pieces of food. Four patients (20%) and one patient (5%) had mild symptoms of bloating and diarrhea (Visick score = II) respectively but the symptoms did not interfere with their life. None of the patients had epigastric pain.

**Table 3:** Gastrointestinal symptoms assessed by Visick classification 1 year after surgery

Visick score	Heartburn	Acid regurgitation	Dysphagia	Bloating	Diarrhea	Epigastric pain
I	20 (100%)	19 (95%)	16 (80%)	16 (80%)	19 (95%)	20 (100%)
II	0	1 (5%)	4 (20%)	4 (20%)	1 (5%)	0
III	0	0	0	0	0	0
IV	0	0	0	0	0	0

The nutritional status before surgery and 1 year after surgery is shown in Table 4. There were no significant changes in total body weight, hemoglobulin, serum total protein, serum albumin or serum prealbumin level postoperatively.

**Table 4:** Body weight and serum nutrition status before surgery and 1 year after surgery

Variable (mean ± sd)	Before surgery	1 year after surgery	*p* value
Total body weight, kg	64.9 ± 10.0	64.4 ± 10.1	0.58
Hemoglobin, g/L	121.1 ± 18.5	124.2 ± 13.4	0.39
Lymphocyte count, 10^9^/L	1.21 ± 0.35	1.38 ± 0.31	0.08
Serum total protein, g/L	65.2 ± 5.4	68.1 ± 6.3	0.09
Serum albumin, g/L	40.2 ± 4.7	41.7 ± 3.3	0.13
Serum prealbumin, g/L	0.23 ± 0.06	0.23 ± 0.04	0.89

## Discussion

PG is regarded as a function preserving procedure for EGC located at the upper-third stomach and EGJ. The retrospective study by Yamashita et al. demonstrated that even for advanced adenocarcinoma of EGJ less than 4 cm in diameter, dissection of the distal LNs of the stomach is not necessary, such as No. 4d, 5, 6 and 12a LNs, arousing surgeons’ great interest for PG [4]. However, the main obstacle for PG is the high incidence of postoperative reflux. Till now, there is no standard procedure for reconstruction after PG [7]. DFT has attracted great attention since its introduction by Kamikawa et al. in 2001 for excellent anti-reflux effect [7, 14]. However, DFT is extremely complicated and time-consuming. SOFY is a relatively simple anti-reflux procedure. But its anti-reflux effect varies among surgeons and patients, with a certain percentage of complications [7, 15]. To overcome the shortcomings of the previous techniques, we designed esophagogastrostomy with ROSF following LPG.

### Efficacy of anti-reflux

For esophagogastrostomy, anti-reflux effect is of great concern. Historically, simple esophagogastrostomy has a high rate of reflux esophagitis (20–65.2%) which prevented surgeons from performing PG. To overcome this, various techniques have been designed, among which DFT had the lowest incidence of reflux esophagitis (0–10.6%) [7]. SOFY was also reported to have lower incidence of reflux esophagitis (7.1–17.8%), but higher than DFT [15]. Table 5 shows the incidence of postoperative complications in patients who underwent ROSF and other reconstruction methods [7, 15–19]. Compared with the these methods, it is encouraging that only one (5%) of our initial 20 patients had mild symptoms of reflux (Visick score = II) and endoscopic reflux esophagitis of LA Grade A was observed in only one patient (5%). However, the incidence of postoperative reflux esophagitis was not significantly different between ROSF and our previous experience of DFT in 23 patients (Table 6). The main mechanisms of anti-reflux of ROSF procedure may fall into two categories. The first is the compression of the anastomotic orifice and lower esophagus by the seromuscular flap. We adopted side overlap with the overlapping length of 3.0 cm, leaving a 5 mm long high-pressure zone in the esophagus above the highest point of the anastomosis. The second mechanism is assumed to be the twist of anastomosis similar to SOFY method. Although our series had low rate of symptomatic and endoscopic reflux, due to limited cases and time of follow-up, the actual performance of ROSF needs further investigation.

**Table 5:** Incidence of complications in reconstruction methods after proximal gastrectomy

Reconstruction method	Anastomotic stenosis (%)	Anastomotic leakage (%)	Reflux esophagitis (%)
EG (ROSF)	0.0	0.0	5.0
EG (DFT)	4.7–29.1	0.0–7.7	0.0–10.6
EG (SOFY)	0.0–2.8	0.0	7.1–17.8
EG (gastric tube)	7.1–28.6	0.0	4.3–30.8
EG (conventional)	0.0–52.2	0.0–18.2	20.0–65.2
JI	3.1–64.3	0.0–13.0	0.0–33.3
JPI	0.0–27.8	0.0–17.2	4.0–27.8
DTR	0.0–20.0	0.0–10.0	4.7–20.0

*EG* Esophagogastrostomy, *ROSF* Right-sided overlap and single-flap valvuloplasty, *DFT* Double-flap technique, *SOFY* Side overlap with fundoplication by Yamashita, *JI* Jejunal interposition, *JPI* Jejunal pouch interposition, *DTR* Double tract reconstruction

**Table 6:** Comparison between Right-Sided Overlap and Single-Flap Valvuloplasty and Double-Flap Technique performed at Second Affiliated Hospital of Soochow University

	**ROSF (*n* = 20)**	**DFT (*n* = 23)**	***p* value**
Surgery time (min), mean ± sd	285.3 ± 71.3	336.5 ± 81.7	0.036
Anastomosis time (min), mean ± sd	61.3 ± 11.2	67.9 ± 9.8	0.046
Anastomotic stenosis, n (%)	0	5 (21.7%)	0.027
Anastomotic leakage, n (%)	0	0	-
Reflux esophagitis, n (%)	1 (5.0%)	4 (17.3%)	0.206

*ROSF* Right-sided overlap and single-flap valvuloplasty, *DFT* Double-flap technique

### Nutritional benefit

The advantage of PG in maintaining postoperative nutritional status has been confirmed by many studies [20]. In our series, the similar benefit was observed (Table 4). At 1-year follow-up, the level of blood nutrition-related parameters did not change significantly, compared to preoperative levels. Similarly, the total body weight 1 year after surgery was not significantly different from the preoperative baseline. The value of ROSF in maintaining postoperative nutrition status of longer follow-up is still to be investigated.

### Advantages in reducing anastomotic complications

Esophagogastrostomy with DFT was reported to have certain incidence of anastomotic stenosis, ranging from 4.7–29.1%, which required balloon dilation [5–7]. The stenosis may develop after several months. On the other hand, SOFY was reported to have lower rate of anastomotic stenosis (0.0–2.8%) [15]. Interestingly, none of our patients had anastomotic stenosis within follow-up period according to Nishikawa’s criterion [12], showing a better effect in preventing anastomotic stenosis than existing methods (Table 5). Compared with our own experience of DFT, anastomotic stenosis decreased significantly in ROSF (DFT: 5, 21.7% vs. ROSF: 0, *p* = 0.027, Table 6). Although four patient (20%) had mild dysphagia (Visick score II), their symptoms relieved simply by adjusting eating habit without any medication or balloon dilation.

The appropriate anastomotic diameter and sufficient blood supply may contribute to the outstanding performance of ROSF in this series. Shoji et al. reported that the diameter of the esophagus < 18 mm was one of the independent risk factors for postoperative anastomotic stricture [5]. The end-to-side esophagogastrostomy may be related to the possibility of stricture, especially in patients with a small lumen of the lower esophagus. Contrarily, overlap esophagogastrostomy is less likely to develop stenosis due to the more spacious anastomosis. In the present study, we utilized overlap anastomosis, ensuring an adequate diameter.

Insufficient blood supply of the seromuscular flap may also contribute to the development of stenosis. Since both flaps are supplied mainly by the right gastroepiploic vessels in DFT, interruption of blood flow by the incision between the flaps might theoretically impair the bloody supply of the right flap. In fact, sometimes we did observe the apparent color change of the right flap after dissection of the flaps when performing DFT. In our study, we speculate that a left-sided single flap may ensure sufficient blood supply to the flap from the left side, which possibly prevents ischemia and consequential stenosis.

Moreover, none of the patients in our study developed anastomotic leakage. Similar to the reasons described above, left-sided single flap may also prevent potential leakage as a consequence of flap ischemia and necrosis, though anastomotic leakage is rare in DFT patients [21, 22] (Tables 5, 6). Theoretically, ROSF may reduce the possibility of leakage better than SOFY, since the anastomosis is covered by the flap, though anastomotic leakage was not reported in SOFY [15]. Based on the considerations above, ROSF might have some advantages in preventing anastomotic stenosis and leakage.

### Simplification of surgical manipulations

While the efficacy of DFT is gradually recognized, technical difficulty and laborious suturing hinders its popularity. In previous studies of DFT, the mean/median surgery and anastomosis time was 235.3–420 and 79.4–109 min respectively [5, 6, 21, 23]. Compared to our previous experience of 23 cases who underwent DFT, the anastomosis time (DFT: 67.9 ± 9.8 min vs. ROSF: 61.3 ± 11.2 min, *p* = 0.046) and overall surgery time (DFT: 336.5 ± 81.7 min vs. ROSF: 285.3 ± 71.3 min, *p* = 0.036) were reduced significantly (Table 6).

In valvuloplasty (both double flap and single flap), closing the flap(s) to cover the esophagus and anastomosis is the most time-consuming procedure. In ROSF, fixing the three brims of the flap with two barbed sutures reduced the complexity and time of suturing. Additionally, in DFT, closing of the flaps is assumed difficult due to lack of anchoring. However, in ROSF, a suture made on the upper-right corner of the flap facilitated the subsequent continuous suturing. Furthermore, overlap anastomosis with linear stapler shortened the time for anastomosis. Though ROSF was shown relatively simple and time-saving in this study, there are still some technical challenges as a seromuscular flap anastomosis method.

Firstly, although cases were not subgrouped according to the length of esophageal invasion, we noticed that performing ROSF in patients with highly-located tumor or short esophagus took more efforts. It needs to be further studied whether ROSF can be easily performed in these patients and what the upper limit of the technique is. Secondly, operation around the esophageal hiatus is difficult due to limited space obstructed by left hepatic lobe. Thus, we applied the liver retraction method shown in Fig. 1A, which provided a satisfactory field of view. However, further studies will be needed to find the optimal liver retraction technique. Lastly, performing ROSF might be hard for surgeons without extensive experience of hand-sewn suturing under laparoscopy, despite that barbed sutures were adopted instead of interrupted sutures. In the future, standardization of the procedure and robotic surgery might contribute to lowering its threshold.

Although ROSF showed benefits in preventing reflux and stenosis and simplifying manipulations, there are some limits of this study. Firstly, this retrospective study had limited number of cases and no comparative analysis. Cohort studies and clinical trials with larger samples are needed to further prove ROSF’s advantages. As a newly introduced technique, follow-up period of ROSF was short. Disease-free survival, long-term complications (such as sliding hernia), and long-term QOL should be investigated in longer time follow-up to confirm its safety and effect. Moreover, 24-h pH monitoring and manometer were not introduced to our institution and thus not conducted in patients. These examinations should be conducted in the future to precisely evaluate postoperative reflux and dysphagia, especially for patients without positive endoscopic results. Lastly, whether ROSF is feasible for all types of adenocarcinoma of EGJ and upper-third stomach needs further investigation.

## Conclusions

In conclusion, we designed a novel method for esophagogastrostomy following LPG, named ROSF. In this case-series study of the initial 20 patients, ROSF showed satisfactory outcomes in terms of preventing reflux and stenosis, nutritional benefits, simplified manipulation, and shortened surgery time. We believe our method is a safe and efficient option for reconstruction after LPG. However, its advantages still require validation in large scale studies with longer follow-up.

## Supplementary Information

**Additional file 1:** **Table S1.** Detailed clinicopathological, surgical and follow-up information of the patients.

## Data Availability

The datasets supporting the conclusions of this article are included within the article and its additional files.

## Abbreviations

ASA-PS: American society of anesthesiologists physical status
BMI: Body mass index
CD: Clavien-Dindo
CECT: Contrast-enhanced computed tomography
DFT: Double-flap technique
DTR: Double tract reconstruction
EG: Esophagogastrostomy
EGC: Early gastric cancer
EGJ: Esophagogastric junction
ESD: Endoscopic submucosal dissection
JI: Jejunal interposition
JPI: Jejunal pouch interposition
LA: Los Angeles
LN: Lymph node
LPG: Laparoscopic proximal gastrectomy
LOS: Length of stay
PG: Proximal gastrectomy
POD: Postoperative day
PPI: Proton pump inhibitor
pStage: Pathological stage
QOL: Quality of life
ROSF: Right-sided overlap and single-flap valvuloplasty
SOFY: Side overlap fundoplication by Yamashita
TG: Total gastrectomy
